# Molecular Response of Simmental Cows to Negative Energy Balance: Regulation of Interleukin-6 and Plasminogen During Early Lactation

**DOI:** 10.3390/ijms262311725

**Published:** 2025-12-03

**Authors:** Kalina Wnorowska, Krzysztof Młynek, Paweł Solarczyk, Beata Głowińska, Karol Tucki, Kamila Puppel

**Affiliations:** 1 Institute of Animal Science and Fisheries, University of Siedlce, ul. B. Prusa 14, 08-110 Siedlce, Poland; 2 Institute of Animal Sciences, Departments of Animal Breeding, Warsaw University of Life Sciences, Ciszewskiego 8, 02-786 Warsaw, Poland; 3 Department of Animal Physiology and Physiotherapy, Faculty of Animal Breeding and Biology, Bydgoszcz University of Science and Technology, 85-796 Bydgoszcz, Poland; 4 Institute of Mechanical Engineering, Department of Production Engineering, Warsaw University of Life Sciences, Nowoursynowska Street 164, 02-787 Warsaw, Poland

**Keywords:** negative energy balance, interleukin-6, plasminogen, Simmental cows, spontaneous lipolysis, β-hydroxybutyrate, leptin, fatty acids, hepatic metabolism, inflammatory response

## Abstract

Negative energy balance (NEB) during early lactation links spontaneous lipolysis (SL) with inflammatory signaling, yet the molecular response in dual-purpose breeds remains insufficiently characterized. This study investigated how NEB regulates circulating concentrations of interleukin-6 (IL-6) and plasminogen (PL) in Simmental cows, contextualizing these changes within concurrent metabolic adaptation. Forty-two cows were monitored from approximately two weeks prepartum to 150 days in milk across six defined stages. Energy balance (EB) was calculated from feed intake and energy-corrected milk yield, while daily milk production (DMP), milk composition, body condition score (BCS), β-hydroxybutyrate (BHBA), glucose (GLU), leptin (LEP), selected fatty acids (FAs: C16:0, C18:0, C18:1-t9, C18:2, IL-6), and PL were determined. EB declined progressively as DMP increased (r = −0.689, *p* ≤ 0.05). During peak NEB (SLII–SLIII), IL-6 increased from 92.16 to 109.59 ng·L^−1^ and PL from 1.65 to 2.05 ng·L^−1^, both inversely correlated with EB (r = −0.741 and −0.586, respectively) and positively associated with each other (r = 0.728), indicating coordinated activation of cytokine and fibrinolytic pathways. NEB severity was accompanied by elevated BHBA and LEP, decreased GLU, reduced BCS, and increased circulating FAs; nevertheless, ketosis remained moderate (peak BHBA 1.04 mmol·L^−1^). These findings demonstrate that Simmental cows display a breed-specific molecular response in which NEB modulates IL-6 and PL in parallel with controlled lipid mobilization and efficient hepatic metabolism, supporting enhanced metabolic resilience during early lactation.

## 1. Introduction

The transition from late gestation to early lactation represents a physiologically demanding and metabolically transitional phase in the life of dairy cows. During this period, the animal undergoes profound metabolic reprogramming to meet the energy demands associated with parturition, uterine involution, and the initiation of milk synthesis. These requirements often exceed dietary energy supply, leading to the development of negative energy balance—a metabolic state in which energy output surpasses intake. This period is characterized by complex biochemical and hormonal adjustments, including enhanced lipolysis and hepatic fatty acid oxidation, increased gluconeogenesis, and dynamic changes in circulating insulin, growth hormone (GH), insulin-like growth factor-1 (IGF-1), leptin, and cortisol concentrations. However, excessive lipid mobilization induces oxidative stress, mitochondrial dysfunction, and adipocyte hypoxia, initiating inflammatory cascades that alter systemic metabolic homeostasis. These responses are orchestrated through molecular signaling networks involving cytokines and lipid mediators [1,2].

The metabolic shift toward lipid catabolism imposes a considerable burden on the liver, the central organ responsible for energy redistribution. Hepatic uptake of NEFA may exceed its oxidative and export capacities, resulting in triglyceride accumulation, impaired β-oxidation, and reduced gluconeogenesis [3,4,5,6]. This dysregulation not only affects hepatic function but also modulates the synthesis of plasma proteins involved in lipid transport, coagulation, and immune response. Consequently, the interplay between lipid metabolism and inflammation represents a key determinant of metabolic stability and disease susceptibility in high-yielding cows.

Among molecular mediators linking lipolysis and inflammation, interleukin-6 and plasminogen are of particular interest. IL-6, a pleiotropic cytokine, regulates both inflammation and metabolism, acting as a central mediator of the acute-phase response and hepatic protein synthesis [7]. During the periparturient period, negative energy balance (NEB) induces metabolic stress that activates a coordinated endocrine–immune response. Among its mediators, interleukin-6 (IL-6) acts as a pleiotropic cytokine that bridges lipid metabolism with inflammatory signaling. IL-6 stimulates hepatic acute-phase protein synthesis through JAK/STAT3 activation and influences lipid turnover by enhancing β-oxidation and modulating very-low-density lipoprotein (VLDL) secretion. Elevated IL-6 concentrations during early lactation are associated with intensified adipose lipolysis, hepatic lipid load, and transient inflammatory activation, reflecting adaptive metabolic remodeling rather than pathological inflammation [8,9]. Plasminogen, traditionally recognized for its fibrinolytic role, also contributes to metabolic adaptation through its involvement in extracellular matrix (ECM) remodeling, cytokine activation, and leukocyte recruitment. Conversion of PL to plasmin via the uPA/uPAR system promotes matrix metalloproteinase (MMP) activation and the release of growth factors that facilitate tissue remodeling and immune–metabolic crosstalk. The parallel regulation of IL-6 and PL suggests a functional link between inflammatory signaling and fibrinolytic activity during NEB, integrating hepatic metabolism, adipose tissue mobilization, and immune adaptation [10,11,12].

Together, IL-6 and PL may serve as molecular biomarkers reflecting metabolic stress, inflammatory activation, and hepatic adaptation during NEB. Recent studies emphasize genetic and physiological determinants of resilience to metabolic stress. Simmental cows are characterized by moderate milk yield, favorable milk composition, and physiological traits associated with greater metabolic stability and adaptability during the transition period [13,14,15]. Their higher hepatic oxidative capacity and lower inflammatory susceptibility make them a valuable model for exploring breed-specific molecular mechanisms of NEB adaptation [16,17].

Despite extensive research on the metabolic and inflammatory consequences of negative energy balance in high-yielding dairy cows, the specific molecular interactions linking lipid mobilization with inflammatory and fibrinolytic pathways remain poorly characterized. Previous studies have primarily focused on Holstein-Friesian cows, whereas data for dual-purpose breeds such as Simmental—recognized for their superior metabolic stability—are scarce. Moreover, the concurrent dynamics of interleukin-6 and plasminogen during the periparturient period have not been systematically quantified in relation to energy balance and metabolic adaptation. The present study aimed to characterize the molecular profile associated with negative energy balance by analyzing dynamic changes in circulating IL-6 and PL as biomarkers of metabolic and inflammatory adaptation. Specifically, we examined how NEB modulates spontaneous lipolysis and the regulation of interleukin-6 and plasminogen—two key mediators linking lipid mobilization and immune activation. By combining physiological, biochemical, and molecular analyses, this study elucidates the molecular response networks governing the adaptive capacity of Simmental cows under NEB, offering mechanistic insight into breed-specific resilience mechanisms that ensure metabolic stability and sustained lactational efficiency. In this study, metabolic resilience refers to the cow’s ability to maintain metabolic homeostasis and recover efficiently from negative energy balance through coordinated regulation of lipid mobilization, hepatic metabolism, and inflammatory signaling. We hypothesized that negative energy balance during early lactation promotes spontaneous lipolysis and activates systemic inflammatory pathways, resulting in elevated circulating concentrations of interleukin-6 and plasminogen. The magnitude and temporal dynamics of these molecular changes were expected to reflect the adaptive metabolic capacity of Simmental cows and their resilience to energetic stress. Furthermore, we anticipated that, compared with high-yielding dairy breeds, Simmental cows would exhibit a more efficient hepatic lipid metabolism and a modulated inflammatory profile, indicative of breed-specific molecular adaptations that support metabolic homeostasis and sustained lactational performance.

## 2. Results

**Figure 1:**
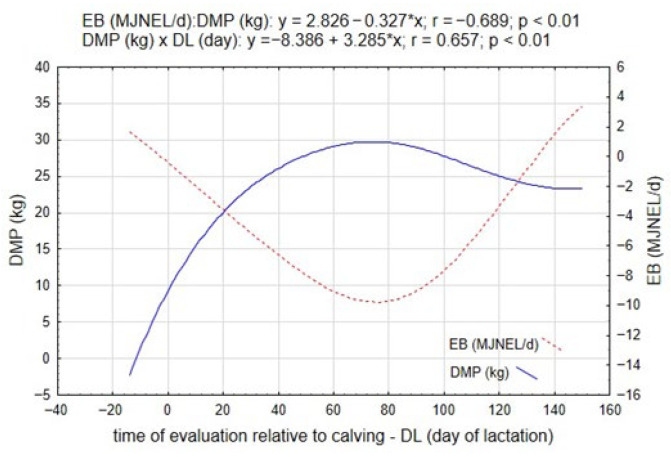
Development of the relationship between daily milk production (DMP) and energy balance (EB) during the experiment.

Figure 1 illustrates the dynamic relationship between daily milk production (DMP) and energy balance throughout the transition period and the early stages of lactation. During the pre-calving period (PCP; approximately 11 days before parturition), EB values ranged from −4.26 to 4.10 MJ NEL/day, with a mean of 1.24 MJ NEL/day (*p* ≤ 0.05; Table 1). This indicates that most cows maintained a near-neutral or slightly positive energy balance before calving.

Following parturition—the onset of the start of the lactation interval (SLI)—a marked divergence between DMP and EB was observed. As lactogenesis intensity increased, DMP rose sharply, accompanied by a progressive decline in EB, reaching its lowest values around the 18th day of lactation (DL). This pattern reflects the characteristic metabolic adaptation to early lactation, during which increasing milk synthesis leads to substantial energy expenditure that exceeds dietary intake. The mathematical relationships derived for these traits (EB = 2.826 − 0.327 × DMP, *r* = −0.689, *p* = 0.01; and DMP = −8.386 + 3.285 × DL, *r* = 0.657, *p* = 0.01) confirm a strong inverse correlation between milk yield and energy balance, and a significant temporal dependency of milk production on days in lactation.

The negative energy balance observed in the early lactation phase demonstrates the intensity of metabolic reprogramming that occurs as the cow transitions from gestation to full lactation. The initial energy deficit is compensated primarily through spontaneous lipolysis (SL) and the mobilization of adipose reserves, processes that support hepatic gluconeogenesis but also predispose to oxidative and inflammatory stress. As lactation progresses beyond approximately 100–120 days, both EB and DMP gradually stabilize, indicating a restoration of metabolic equilibrium and improved synchrony between energy intake and output.

As shown in information contained in Section 4.3, the chemical composition and energy value of the diet were comparable between the two studied farms. The average dry matter content was 42.4% and 41.6%, while crude protein ranged from 16.9 to 17.5%, and fat content remained low (2.3–2.5%). Fibre fractions were balanced across both feeding systems (ADF ≈ 22%, NDF ≈ 39%), providing adequate rumen function. The calculated UFL values (21.4–21.6) and protein digestibility indices (PDIN ≈ 2500 g, PDIE ≈ 2190 g) confirmed that the nutritional level met the requirements for mid-lactation dairy cows.

Energy balance was slightly positive on both farms, averaging +1.4 MJ NEL/day and +1.7 MJ NEL/day, indicating a well-balanced diet under the given feeding regime. However, during the early post-calving period, a transient decline in EB was observed, reaching a mean decrease of −3.9 MJ NEL/day (*p* ≤ 0.05), concurrent with a significant increase in milk yield. This trend persisted until approximately 76 days in lactation (DL) (Figure 1). Over this period, daily milk production (DMP) increased by an average of 5.0 kg (*p* ≤ 0.05), while the energy deficit deepened by an additional −4.6 MJ NEL/day (*p* ≤ 0.05).

The progressive increase in milk yield was strongly and negatively correlated with energy balance (r = −0.689, *p* ≤ 0.05), indicating that higher milk production was associated with a greater energy deficit.

In the context of dietary management and the mitigation of negative energy balance, both milk yield and the concentrations of major milk constituents were closely monitored (Table 1). The results reveal that during the initial stages of lactation (SLI–SLIII), corresponding to the period of deepest energy deficit, milk exhibited lower levels of protein, fat, lactose, and total solids. In contrast, during SLIV and SLV, when cows gradually restored energy equilibrium, the concentrations of these components increased significantly.

The smallest variation was recorded for protein content (Δ = 0.08%; *p* ≤ 0.05), and the correlation between total protein and EB was weak (r = 0.245, *p* ≤ 0.01), suggesting that protein synthesis was relatively stable despite energy fluctuations. In contrast, fat and lactose contents demonstrated more pronounced differences, amounting to 0.22% and 0.53%, respectively (*p* ≤ 0.05). These components were more strongly associated with EB, as evidenced by correlation coefficients of r = 0.517 (fat) and r = 0.696 (lactose) (*p* ≤ 0.01), indicating a direct metabolic link between energy status and mammary gland activity.

Moreover, the content of dry matter (r = 0.603, *p* ≤ 0.01) showed a significant positive relationship with EB, reflecting the combined influence of lipid and carbohydrate fractions on milk composition. The pattern of variation in these milk components closely mirrored the dynamics of EB presented in Figure 1, confirming that the magnitude of negative energy balance induced by lactogenesis directly affects the biosynthesis and secretion of key milk constituents. Overall, the data suggest that energy deficit primarily alters the metabolic prioritization of substrates in the mammary gland

Table 2 presents the temporal changes in key metabolic biomarkers used to characterize the physiological consequences of negative energy balance in Simmental cows. Between the pre-calving period (PCP) and the start of lactation interval (SLI)—when a progressive decline in energy balance (EB, MJ NEL/day) was recorded (Figure 1)—only minor variations were observed in the blood concentrations of glucose (GLU) and leptin (LEP). The differences amounted to 0.08 mmol·L^−1^ (*p* ≤ 0.05) for GLU and 0.09 ng·mL^−1^ for LEP, and were not statistically significant. Similarly, β-hydroxybutyrate (BHBA) concentration remained stable, with a mean difference of 0.05 mmol·L^−1^, indicating that major metabolic adjustments had not yet been triggered at this stage of the transition period.

The most pronounced alterations in biomarker concentrations occurred during the intensive milk production phase (SLI–SLII), corresponding to the onset of peak lactogenesis and the deepest NEB (Figure 1). During this period, BHBA concentration increased from 0.52 to 0.89 mmol·L^−1^ (*p* ≤ 0.05), reflecting enhanced hepatic ketogenesis due to the mobilization of non-esterified fatty acids from adipose tissue. Simultaneously, leptin concentration rose by 0.19 ng·mL^−1^ (*p* ≤ 0.05), which is consistent with adipocyte stimulation during lipolysis and the involvement of LEP in metabolic signaling associated with energy deficiency. In contrast, glucose concentration decreased significantly by 0.25 mmol·L^−1^ (*p* ≤ 0.05), indicating intensified glucose utilization by mammary epithelial cells and limited hepatic gluconeogenic capacity. Beyond the peak production stage, the dynamics of these biomarkers gradually stabilized. From SLIII to SLV, the concentrations of GLU and LEP exhibited only minor and statistically non-significant fluctuations, indicating partial restoration of energy balance and metabolic homeostasis. A marked reduction in daily milk production by approximately 5.2 kg (*p* ≤ 0.05) (Table 1) coincided with this stabilization, confirming the coupling between lactogenesis intensity and metabolic load.

Specifically, BHBA concentration declined from its maximum at SLIII to 0.90 mmol·L^−1^ in SLIV and 0.69 mmol·L^−1^ in SLV, representing decreases of 0.14 and 0.21 mmol·L^−1^, respectively (*p* ≤ 0.05). This reduction reflects lower ketone body synthesis as lipid mobilization subsided. A similar trend was noted for LEP, whose level decreased significantly by 0.12 ng·mL^−1^ (*p* ≤ 0.05) in SLV, suggesting normalization of adipocyte activity with improved EB. Conversely, glucose concentration began to rise following the production peak (SLIII), increasing by 0.09 mmol·L^−1^ in SLIV (*p* ≤ 0.05), and then remaining stable throughout the late lactation phases.

Overall, these data demonstrate that the dynamics of BHBA, GLU, and LEP concentrations are closely synchronized with the intensity of lactogenesis and the depth of NEB. Elevated BHBA levels and reduced glucose availability indicate a metabolic shift toward lipid oxidation and ketogenesis, while changes in leptin reflect adaptive regulation of energy intake and substrate mobilization.

The observed trends in the blood concentrations of the analyzed biomarkers (Table 2) and the concurrent variations in energy balance (EB, MJ NEL/day) presented in Figure 1 demonstrate a strong association between these parameters. This conclusion is supported by the correlation coefficients listed in Table 2. The most pronounced relationship was observed between EB and β-hydroxybutyrate concentrations (r = −0.872; *p* ≤ 0.01), confirming that the intensification of lipolytic processes and hepatic ketogenesis is tightly coupled with the degree of negative energy balance. A similar, though slightly weaker, negative correlation was found between EB and leptin (r = −0.562; *p* ≤ 0.01), indicating that adipose tissue activity and leptin secretion are also responsive to fluctuations in the energy status of the organism. In contrast, a moderate but statistically significant positive correlation was observed between EB and GLU concentration (r = 0.579; *p* ≤ 0.01), suggesting that improvements in energy balance correspond with restored glycemic stability and hepatic gluconeogenic efficiency.

**Figure 2:**
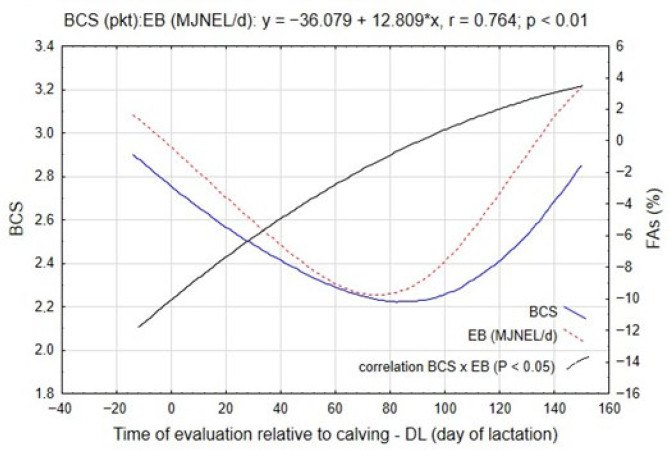
Trends in body condition score and total fatty acids during the experiment, along with their correlation (black line).

One of the physiological indicators reflecting the intensity of lactogenesis and metabolic adaptation is the body condition score of cows. The dynamics of BCS in relation to EB changes are presented in Figure 2. The data indicate that the variation in BCS closely mirrored the temporal pattern of EB. During the pre-calving period, approximately 11 days before parturition, BCS values ranged between 2.5 and 3.2 points, reflecting an adequate energy reserve prior to calving. According to Table 3, the transition into early lactation (SLI; approximately the 18th day of lactation) was characterized by the most pronounced reduction in BCS, averaging 0.28 points (*p* ≤ 0.05), consistent with the deepening NEB observed during this interval.

This downward trend persisted until approximately the 76th day of lactation (SLIII), with successive decreases averaging 0.20 points (*p* ≤ 0.05) between intervals. The reduction in BCS during this phase is indicative of intense adipose tissue mobilization, which provides substrates for hepatic oxidation and supports milk synthesis during peak lactogenesis. As shown in Figure 2, a slight improvement in BCS was detected after the production peak (SLIII; approximately the 107th day of lactation), amounting to 0.08 points, although this change was not statistically significant. A more evident recovery occurred in the later stage of lactation (SLV), with an average increase of 0.42 points (*p* ≤ 0.05), confirming the partial restoration of energy equilibrium and the reduction in lipid catabolism as feed intake surpassed energy expenditure.

The results presented in Table 3 demonstrate that energy balance (EB, MJ NEL/day) exerted a significant influence on the dynamics of body condition score and circulating FAs concentrations in Simmental cows. This effect was mediated by the intensity of lipolytic activity, which increased proportionally with the deepening of negative energy balance. The pattern of these relationships, illustrated in Figure 2, was confirmed by a strong positive correlation between EB and BCS (r = 0.764; *p* ≤ 0.01), indicating that energy deficit directly drives body reserve mobilization through enhanced lipolysis.

Between the pre-calving period and the start of lactation interval, BCS decreased markedly, from an average of 2.88 to 2.60 points (*p* ≤ 0.05). This reduction reflected the initiation of adipose tissue catabolism necessary to compensate for the growing energy demands associated with parturition and the onset of lactogenesis. The accompanying increase in FAs concentrations during this stage supports this conclusion. The proportions of C16:0 (palmitic acid), C18:0 (stearic acid), C18:1-t9 (trans-oleic acid), and C18:2 (linoleic acid) increased by 0.7–1.2% (*p* ≤ 0.05), confirming the mobilization of long-chain fatty acids from adipocytes into circulation (Table 3).

During SLII, when the mean BCS reached 2.40 points (*p* ≤ 0.05), the concentrations of all analyzed fatty acids exhibited their maximum values, with the most prominent increases recorded for C16:0 (+1.8%; *p* ≤ 0.05) and C18:0 (+0.4%; *p* ≤ 0.05) relative to SLI. These data indicate the period of highest lipolytic intensity, coinciding with the lowest energy balance values and peak lactogenesis (Figure 1).

In the subsequent intervals (SLIII–SLIV), a stabilization of FAs concentrations was observed, reflected in smaller, statistically non-significant differences between periods. This trend suggests attenuation of lipolytic activity as energy balance gradually improved and dietary intake began to meet energy requirements. By SLV, both FAs concentrations and BCS exhibited recovery patterns: BCS increased significantly from 2.27 to 2.69 points (*p* ≤ 0.05), while the concentrations of major FAs—particularly C16:0 and C18:0—returned close to their pre-calving levels. These observations indicate a transition from lipid mobilization to lipid re-synthesis, marking the restoration of metabolic equilibrium (Table 3).

The relationships between EB and individual fatty acids were strongly negative, confirming the dependence of lipolysis on energy status. The correlation coefficients ranged from r = −0.849 (*p* ≤ 0.01) for C16:0 to r = −0.659 (*p* ≤ 0.01) for C18:2, indicating that decreases in EB were consistently associated with elevated circulating FAs concentrations. Furthermore, a significant negative correlation was found between BCS and the total FAs pool (r = −0.759; *p* ≤ 0.01).

Collectively, these results confirm that energy balance, body condition, and FAs acid mobilization are tightly interconnected components of the metabolic adaptation to early lactation. The observed increase in saturated (C16:0, C18:0) and unsaturated (C18:1-t9, C18:2) fatty acids reflects the metabolic prioritization of lipid catabolism to support hepatic gluconeogenesis and mammary energy requirements.

**Figure 3:**
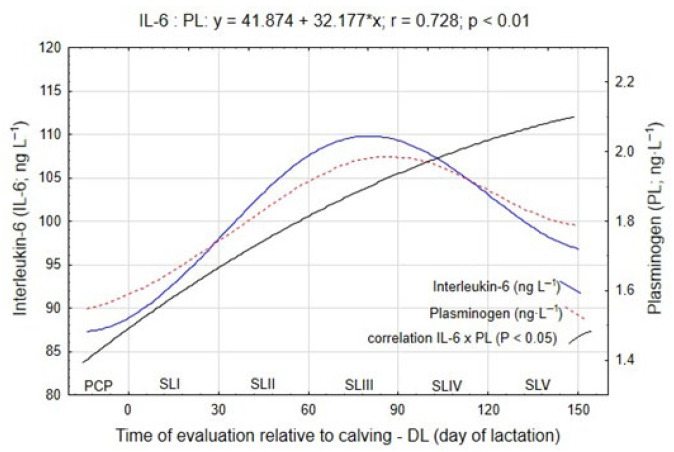
Dynamics of pro-inflammatory marker concentrations in the blood of cows depending on the experimental period.

The metabolic processes initiated by lipolysis in adipocytes can contribute to the development of systemic and local metabolic dysfunctions. In the present study, special emphasis was placed on analyzing the dynamics of lipolysis and its impact on the induction of inflammatory responses within adipose tissue. Table 4 summarizes the changes in the concentrations of two pro-inflammatory proteins—interleukin-6 and plasminogen—measured during consecutive lactation stages, while Figure 3 illustrates the temporal dynamics of these biomarkers in relation to energy balance (EB, MJ NEL/day).

Between the pre-calving period (PCP) and the start of lactation interval, both IL-6 and PL concentrations remained relatively stable, averaging 89.88 ng·L^−1^ and 1.62 ng·L^−1^, respectively. During this period, cows maintained near-neutral EB values, and no significant inflammatory activation was observed. However, a pronounced rise in the concentrations of both biomarkers occurred during SLII, coinciding with the phase of deepest negative energy balance (NEB) and the most intense spontaneous lipolysis (Figure 1). Compared with the SLI stage, IL-6 increased by 11.6 ng·L^−1^ (*p* ≤ 0.05), while PL rose by 0.14 ng·L^−1^ (*p* ≤ 0.05).

A further significant increase in plasminogen concentration was recorded in SLIII, reaching 2.05 ng·L^−1^, representing a difference of 0.26 ng·L^−1^ (*p* ≤ 0.05) compared with the earlier stage. These results indicate that as lipolysis intensifies under NEB conditions, the activation of inflammatory pathways in adipose tissue also increases.

During SLIV, concentrations of IL-6 and PL stabilized, indicating a partial attenuation of the inflammatory response as energy balance gradually improved. Subsequently, in SLV, both markers showed a decline in concentration, decreasing by 7.71 ng·L^−1^ (IL-6) and 0.15 ng·L^−1^ (PL) (*p* ≤ 0.05), suggesting that as lactation progressed and NEB resolved, inflammatory activity subsided and systemic homeostasis was restored.

Statistical analysis confirmed a strong negative correlation between EB and both IL-6 and PL concentrations, with correlation coefficients of r = −0.741 and r = −0.586, respectively (*p* ≤ 0.01), indicating that decreases in energy balance were associated with higher levels of inflammatory mediators. During peak NEB, IL-6 and PL concentrations increased synchronously and were negatively correlated with energy balance (r = −0.741 and −0.586, respectively) and positively correlated with each other (r = 0.728). The parallel up-regulation of IL-6 and PL indicates a coordinated regulatory relationship linking lipid mobilization with inflammatory control, which may represent a molecular hallmark of metabolic resilience in this dual-purpose breed (Figure 3).

## 3. Discussion

The transition from gestation to lactation constitutes one of the most metabolically demanding and biologically dynamic phases in the life cycle of dairy cows. During this period, the mammary gland undergoes extensive metabolic and molecular reprogramming to support the onset of lactogenesis. The capacity to sustain energy homeostasis depends on the efficient coordination of catabolic and anabolic pathways, primarily regulated through the availability of metabolizable energy (EB, MJ NEL/day). This energy flux governs not only the intensity of milk synthesis but also the integration of hepatic, adipose, and endocrine networks required to maintain physiological stability under the challenge of negative energy balance. Effective lactation thus reflects a balance between metabolic pressure and the activation of adaptive molecular mechanisms that enable cows to offset transient energetic deficits without compromising systemic integrity.

The present results demonstrate a pronounced influence of energy balance on milk composition in Simmental cows. Among the major milk constituents, lactose (r = 0.696; *p* ≤ 0.01) and fat (r = 0.517; *p* ≤ 0.01) displayed the strongest positive associations with EB, while total protein content exhibited only a weak correlation (r = 0.245; *p* ≤ 0.01). This pattern suggests that, under NEB, the overall protein concentration of milk reflects systemic metabolic status rather than mammary biosynthetic activity. Earlier studies by Darewicz et al. [18] and Falta et al. [19] reported similar trends, showing that during energy deficit, the relative proportion of whey proteins—mainly albumins and immunoglobulins—rises due to translocation from plasma, driven by increased epithelial permeability and altered tight-junction integrity. These proteins, entering milk through paracellular leakage or transcytosis, serve as indicators of systemic immune activation rather than enhanced secretory function. The concordance of these findings with our data reinforces the view that total milk protein concentration is not a reliable proxy for mammary synthetic capacity during metabolic stress but rather an integrative biomarker of whole-body homeostatic disruption.

The interplay between metabolic and inflammatory signaling was further reflected in the dynamics of leptin and interleukin-6. Both molecules exhibited significant negative correlations with EB (LEP: r = −0.562; IL-6: *r* = −0.741; *p* ≤ 0.01), underscoring their role as sensitive molecular mediators of the lipolytic–inflammatory interface. Leptin, secreted by adipocytes, transiently decreased in the periparturient period—facilitating increased feed intake—then rose as lipolysis intensified, reflecting the activation of adipose tissue and the initiation of pro-inflammatory signaling. IL-6, in turn, acts as a pleiotropic cytokine that integrates lipid metabolism with the hepatic acute-phase response. Its increase during peak NEB indicates the engagement of immune–metabolic pathways that preserve energy redistribution and protect against excessive lipid accumulation in the liver. IL-6 signals through IL-6R (membrane or soluble) and the gp130 co-receptor to activate JAK/STAT3 (with context-dependent MAPK/ERK and PI3K inputs), directly inducing hepatic acute-phase genes (e.g., SAA, fibrinogen, haptoglobin) and hepcidin via STAT3 promoter binding, thereby coupling inflammatory tone with liver protein synthesis [20]. In hepatocytes, IL-6 can also remodel lipid flux: experimental IL-6 administration reduces steatosis by increasing mitochondrial fatty acid oxidation and enhancing VLDL export, indicating an upstream influence on hepatic NEFA handling during high lipid load states [21]. While chronic IL-6 may promote steatosis/fibrosis in other contexts, in acute metabolic stress it supports hepatocyte homeostasis and regeneration—consistent with a time- and dose-dependent, “double-edged” hepatic role [22]. Beyond fibrin clearance, the plasminogen–plasmin axis orchestrates leukocyte recruitment, cytokine bioavailability, and ECM turnover. Plasmin generated via uPA/uPAR cleaves ECM, activates latent MMPs, and liberates matrix-bound growth factors, facilitating immune-cell trafficking and tissue [23]. Immune cells express distinct plasminogen receptors (e.g., Plg-RKT, annexin A2), positioning plasmin activity at the cell surface to regulate phagocytosis/efferocytosis and resolution of inflammation [24]. Mechanistic crosstalk may occur at two levels. First, transcriptional coupling: IL-6/STAT3–C/EBPβ signaling in hepatocytes can up-regulate plasminogen expression as part of the acute-phase program, potentially coordinating cytokine and fibrinolytic capacity during NEB [25]. Second, functional coupling: IL-6 promotes an acute-phase milieu and metabolic reprogramming in the liver, while the PL/uPA/uPAR system remodels ECM and gates leukocyte migration; together they align hepatic lipid processing with peripheral tissue remodeling and immune trafficking—providing a plausible mechanistic basis for the correlated IL-6 and PL dynamics we observed under peak NEB. Importantly, the concurrent elevation of IL-6 and plasminogen observed in our study demonstrates that these factors are not merely passive biomarkers of stress, but active molecular regulators within an adaptive signaling network. Their parallel dynamics suggest a functional regulatory axis linking adipose tissue lipolysis, hepatic metabolic remodeling, and fibrinolytic system activation. This coordinated molecular response may serve to maintain circulatory and tissue homeostasis during high metabolic load. Such coupling between cytokine release and fibrinolytic activity delineates a conserved adaptive pathway in Simmental cows that integrates immune, metabolic, and vascular regulation under NEB.

Changes in body condition score and fatty acid profiles further substantiate the metabolic adaptations observed. The progressive decline in BCS from the pre-calving period through SLIII corresponded with increased plasma concentrations of C16:0, C18:0, C18:1-t9, and C18:2, indicative of intensified adipose tissue lipolysis. Consistent with reports by Roche et al. [26] and Grummer [27], reduced BCS was accompanied by elevated non-esterified fatty acids and β-hydroxybutyrate. However, in Simmental cows, BHBA concentrations remained moderate, suggesting efficient hepatic β-oxidation and enhanced lipid clearance capacity, which collectively reduce the risk of ketosis. These findings align with Sordillo and Raphael [28], who proposed that moderate-producing breeds display a more balanced metabolic phenotype, characterized by optimized lipid oxidation and reduced oxidative overload.

Previous studies have reported that sustained lipolysis can increase mitochondrial β-oxidation and reactive oxygen species (ROS) production, potentially activating cytokine and immune signaling pathways [29,30]. Although oxidative stress markers were not assessed in this study, this mechanism may provide a physiological context for the observed associations between fatty acid mobilization, IL-6, and plasminogen, reflecting low-grade inflammatory activation within adipose tissue. Previous research [31,32,33] similarly linked chronic NEB with cytokine-driven inflammation (IL-6, TNF-α) and immune dysregulation. Beyond its canonical fibrinolytic role, plasminogen may act as a signaling modulator, amplifying IL-6-dependent acute-phase cascades and facilitating extracellular matrix remodeling during metabolic stress. Together, IL-6 and PL appear to form a dual regulatory module that coordinates metabolic stress responses with inflammatory control, representing a molecular framework underlying metabolic resilience in Simmental cows. Breed-specific comparisons further reinforce this interpretation. Studies by Strączek et al. [34] and Gross et al. [35] reported stronger correlations between IL-6, plasminogen, and NEB severity in Holstein-Friesian (HF) cows, consistent with their higher metabolic turnover and limited adaptive flexibility. In contrast, Simmental cows maintained a modulated inflammatory and metabolic profile, with lower cytokine peaks and superior hepatic lipid clearance [14,15,36]. Simmental cows maintained a modulated inflammatory and metabolic profile, with lower cytokine peaks and superior hepatic lipid clearance. In Holstein-Friesian cows under comparable early-lactation NEB, mean plasma IL-6 concentrations reach approximately 98.9 ng·L^−1^ and plasminogen about 2.45 ng·L^−1^, whereas in Simmental cows these values were 109.6 ng·L^−1^ and 2.05 ng·L^−1^, respectively [9]). This comparison indicates that despite slightly higher circulating IL-6, Simmental cows exhibited more efficient hepatic lipid turnover and limited ketone body accumulation, supporting a breed-specific adaptive mechanism that restrains inflammatory activation while sustaining metabolic flexibility. This supports the concept of breed-specific molecular adaptation, where moderate-yielding cows rely on more stable cytokine signaling and oxidative balance to mitigate NEB-induced stress. Genetic and transcriptomic data [37,38] indicate that such differences may originate from variable expression of genes controlling immune–metabolic and fibrinolytic pathways. Proteolysis also contributes to this adaptive circuitry. The elevated plasminogen observed during early lactation may represent a compensatory mechanism for protein deficiency under restricted intake, consistent with cortisol-mediated activation of proteolytic genes [31]. These processes illustrate the integration of endocrine stress responses into the broader molecular framework of NEB adaptation. Additionally, prolactin emerges as a key hormonal regulator that couples lactogenic drive with lipid metabolism and energy balance [39,40]. In high-producing breeds, hyperprolactinemia may prioritize milk synthesis over metabolic recovery, while in Simmental cows, lower PRL signaling likely allows a more harmonized regulation of lipogenesis, lipolysis, and leptin expression [41]. This endocrine balance contributes to their superior capacity for homeostatic restoration post-calving. Recent transcriptomic studies in humans [42,43] revealed that genes governing lipid metabolism in mammary tissue are tightly co-regulated with immune pathways. Similar gene–metabolite interactions likely occur in cattle, where coordinated expression of leptin-, FAs-, and cytokine-related genes may define the degree of adaptation to NEB. Such molecular coordination may underlie the resilience phenotype observed in Simmental cows—characterized by moderate production intensity, efficient hepatic lipid turnover, and controlled inflammation.

Despite advances in our understanding of energy metabolism, the molecular networks that orchestrate adaptive responses to NEB remain incompletely elucidated. Future research integrating metabolomic, transcriptomic, and proteomic approaches will be essential to unravel these mechanisms. Collectively, the present findings provide compelling molecular evidence that Simmental cows respond to NEB through an integrated regulatory axis coupling lipolysis, cytokine production, and hepatic metabolism. This orchestrated molecular response ensures effective energy redistribution while preventing uncontrolled inflammation, constituting a robust physiological model of metabolic resilience and molecular self-regulation that may inform future breeding and nutritional optimization strategies in dairy cattle. Building on the identified IL-6–plasminogen regulatory axis, future work should test practical interventions that align with Simmental metabolic resilience. Nutritional candidates include: (i) rumen-protected choline and methionine to support VLDL assembly/export (APOB/MTTP) and mitigate hepatic lipid load; (ii) niacin to temper excessive lipolysis (PNPLA2/LIPE) and lower NEFA influx; (iii) omega-3 fatty acids (EPA/DHA) to moderate IL-6/STAT3 activity and cytokine tone; and (iv) propylene glycol peri-calving to bolster gluconeogenic supply and limit ketone formation. From a genetics perspective, genomic selection anchored to variants near PPARA, CPT1A, PLG/PLAU/PLAUR may enrich for the hepatolipid–fibrinolytic profile associated with low ketosis risk. Mechanistic validation should include CRISPR perturbation in bovine hepatocyte/adipocyte models (targeting STAT3/CEBPB, PPARA, PLG/PLAU/PLAUR) to confirm causal links while respecting on-farm regulatory constraints. These focused pathways and actionable levers provide a concrete roadmap for translating the Simmental phenotype into scalable, welfare-friendly management gains.

## 4. Materials and Methods

### 4.1. Housing Conditions and Health of Cows

The cows had a mean body weight of 670 ± 38 kg (range: 610–730 kg) at the beginning of the study. The experimental group included 14 cows in their second parity, 16 in their third parity, and 12 in their fourth parity. The average milk yield from the previous lactation was 7450 ± 480 kg, with mean milk composition values of 4.05% fat, 3.42% protein, and 4.80% lactose. All cows were clinically healthy, maintained under uniform feeding and management conditions, and represented typical performance levels for the Simmental dual-purpose population under commercial production systems.

The experiment was conducted on 42 multiparous Simmental dairy cows maintained on two commercial farms under standard production conditions located in the Masovian region of Poland. Both farms followed comparable management systems, including free-stall housing, twice-daily milking, and similar feeding strategies based on total mixed rations (TMR). The experimental procedures, sampling schedule, and analytical protocols were standardized across farms to ensure methodological consistency. All animals were housed in a free-stall system, forming a single feeding group with ad libitum access to the resting and feeding areas. The housing environment consisted of individual cubicles with straw bedding, a common feed table, and freely accessible water troughs. The number of stalls and the surface area of each functional zone met the standards specified in the Regulation of the Minister of Agriculture and Rural Development of 28 June 2010 on the minimum requirements for the maintenance of farm animal species other than those for which welfare standards are defined in the European Union legislation (consolidated text, OJ 2017, item 127).

All experimental procedures were performed in accordance with animal welfare and ethical standards applicable to dairy cattle management.

### 4.2. Experiment Layout

The experiment began approximately two weeks before the expected calving date and continued until the cows exhibited measurable recovery of body condition score. The observation period was divided into six experimental stages. The first stage represented the pre-calving period (PCP), followed by five defined lactation stages (SL), corresponding to specific days in milk (DIM):

- SLI: 5–30 DIM
- SLII: 31–60 DIM
- SLIII: 61–90 DIM
- SLIV: 91–120 DIM
- SLV: 121–150 DIM

During the PCP, each cow was evaluated once, approximately 14 days before the predicted calving date. The first post-partum sampling was conducted on day 4 after calving, corresponding to the colostrum secretion phase, to capture the onset of metabolic and physiological changes associated with early lactation.

To minimize animal stress, ensure regular sampling across the herd, and maintain precision in analyzing the dynamics of negative energy balance markers, the cows were organized into six subgroups of seven animals each, randomly selected from the herd. These subgroups remained constant throughout the study. Each subgroup was examined sequentially during sampling sessions, preserving a consistent order of assessment. Sampling was conducted five times during SLI and six times during SLII–SLV, at approximately five-day intervals within each stage. Consequently, data for each cow were collected approximately every 30 days over the course of the experiment.

### 4.3. Control of the Diet and Body Conditions of Cows

Nutritional requirements were estimated based on feed composition, chemical analyses, average body weight, and projected milk yield for each lactation stage, in accordance with AOAC [44] standards. The nutritional value of feed was determined immediately before each sampling stage, and analyses included chemical composition and particle size distribution, ensuring uniformity and precision in dietary control.

During the pre-calving period, cows received a preparatory diet formulated to support metabolic adaptation to the upcoming lactation. Body condition score was assessed at every stage of the study using a standardized five-point scale and served as one of the primary indicators of lipolytic intensity and NEB progression. This approach enabled parallel evaluation of energy status, milk production dynamics, and metabolic biomarkers throughout the experimental period.

Nutritional requirements were estimated based on feed composition, chemical analyses, average body weight, and projected milk yield for each lactation stage, in accordance with the INRA nutritional guidelines for ruminants. Diet formulation was performed using the INRAtion software (version 2.xx; National Research Institute of Animal Production, Kraków, Poland). The total mixed ration (TMR) was adjusted to meet the requirements for metabolizable energy and protein according to AOAC (2007) standards for analytical procedures [45]. The cows were fed a total mixed ration (TMR), in which all feed components were thoroughly homogenized in a feed wagon before distribution. The ration was supplied three times daily at approximately 8 h intervals, with regular feed push-ups performed to ensure continuous feed availability and minimize sorting behavior.

The basal diet was formulated for cows with an average body weight of 650 kg and a target milk yield of 25 L/day. For cows exceeding this production level, an additional concentrate supplement was provided to meet increased energy and protein demands. The mean quantity of feed refusals was determined by periodic weighing, performed twice monthly during each experimental stage, and was used to calculate dry matter intake (DMI). All dietary components—including forages, concentrates, and supplements—were incorporated into the DMI estimations.

The diet composition remained consistent across both herds. The average nutrient content of the daily ration used throughout the study is summarized in Table 5, while Table 6 lists the ingredients that constituted the cows’ daily diet. The formulated rations were designed to meet the nutritional requirements for mid- to high-yielding dairy cows, ensuring optimal energy balance, rumen function, and metabolic stability across all stages of lactation.

Energy balance was calculated as the difference between metabolizable energy intake and the energy expended for milk production and maintenance. The energy content of milk was adjusted for composition (fat, protein, and lactose) according to the formula proposed by Sjaunja et al. [46]. Thus, EB was determined as:

EB = E_intake_ − E_milk_

where E_intake_ represents the daily metabolizable energy intake derived from feed, and E_milk_ denotes the total energy required for milk synthesis and physiological maintenance during lactation. Energy output in milk (E_milk_) was expressed in megajoules (MJ) using the conversion factor of 4.19 J/cal, as described by Taylor [47]. E_intake_ was estimated from the mean dry matter intake (DMI) recorded during each analyzed period, multiplied by the energy content of one kilogram of the total mixed ration (TMR) (Table 1).

The net energy requirement for maintenance was calculated from metabolic body weight (BW^0.75^ × 0.08), and the energy used for milk production was calculated according to the National Research Council [48] equation:

E_milk_ = (0.0929 × %fat) + (0.0588 × %protein) + (0.0395 × %lactose)

All EB values were expressed in megajoules of net energy for lactation per day (MJ NEL/day).

### 4.4. Collection of Material for Analysis

The collection of analytical material followed the experimental schedule described in Section 4.2. Throughout the study, the amount of uneaten feed (refusals) was recorded for each feeding cycle. Refusals were weighed immediately before the next feeding, and the average mass of feed refusals for the herd was calculated on sampling days, approximately every five days. These data were subsequently incorporated into the calculation of energy balance (EB), as detailed in Section 4.3.

During the pre-calving period, body condition score was assessed based on the estimated calving date. In each subsequent lactation stage, BCS was evaluated once per subgroup. The body condition score (BCS) was assessed using the BCS-5 method described by Wildman et al. [49], which evaluates the fat reserves based on visual and manual inspection of specific anatomical sites, including the ribs, spine, and pelvis. The scale ranges from 1 to 5, with 1 indicating severe emaciation and negligible fat reserves, and 5 representing excessive adiposity with prominent fat deposits throughout the body. The evaluation was conducted by trained assessors to ensure consistency and objectivity.

During all SL stages, daily milk production (DMP) was recorded as the total volume obtained from morning and evening milkings. Concurrently, milk samples were collected for compositional analysis. Approximately 250 mL of milk was collected using a Tru-Test Datamars milk meter (New Zealand), ensuring proportional representation of the entire milking volume (approximately 1 mL of sample per 1 L of yield). The individual portions were pooled to create a composite control sample representative of each cow. All milk samples were immediately stored under refrigerated conditions (4 °C) until further laboratory analysis to maintain compositional integrity.

Blood sampling was conducted from the subcutaneous abdominal (mammary) vein before the morning feeding to minimize metabolic variability and circadian interference. This sampling site was selected based on its physiological relevance to mammary metabolism: blood leaving the udder through the mammary vein carries locally derived metabolites, cytokines, and fibrinolytic mediators reflecting the post-mammary microenvironment. This approach allowed detection of local molecular interactions between lipid mobilization and inflammatory signaling that are often attenuated in systemic venous blood. Furthermore, the mammary vein was chosen to ensure methodological consistency with arterio-venous sampling studies describing metabolic gradients across the mammary gland [50,51,52]. All procedures were approved by the Institutional Ethics Committee (approval no. 70/2013) and were performed during routine milking without additional restraint to minimize animal stress and avoid stress-induced cytokine fluctuations that may occur with repeated jugular venipuncture. Samples were collected into disposable vacuum tubes (MedLab-Products, Pruszków, Poland). Blood intended for biochemical and hormonal analyses was drawn into tubes containing EDTA as an anticoagulant to obtain plasma, while samples for glucose (GLU) determination were collected into tubes coated with sodium fluoride to inhibit glycolysis. After centrifugation, plasma was separated within 10 min of rotor stop to minimize analyte degradation. The plasma was aliquoted immediately into labeled polypropylene tubes and stored at −20 °C until biochemical analyses or at −80 °C for cytokine and plasminogen assays. All aliquots were processed under identical pre-analytical conditions, and no additional treatments were applied to the samples used for different assays. To prevent hemolysis, all samples were gently mixed and immediately placed in a portable refrigerator (CDF18; DanLab, Białystok, Poland) at +4 ± 1 °C. Blood destined for glucose analysis was transported directly on ice to further reduce post-sampling glucose metabolism. All samples were processed promptly to ensure analytical reliability.

### 4.5. Analytical Procedures

The basic composition of milk (fat, protein, and lactose content) was determined using a Bentley Combi 150 analyzer (Bentley Instruments Inc., Chaska, MN, USA). Only samples collected from cows free of inflammatory udder conditions were included in the analysis. Health status was verified based on microbiological quality criteria consistent with Regulation (EC) No. 853/2004 of the European Parliament and of the Council of 29 April 2004, which defines hygiene standards for food of animal origin. Specifically, only milk with a somatic cell count (SCC) ≤ 400,000 cells/mL and a plate count at 30 °C ≤ 100,000 CFU/mL was accepted for chemical evaluation.

#### Blood Preparation and Biomarker Determination

Blood samples were processed to obtain plasma through centrifugation (MPW-352RH centrifuge, DanLab, Białystok, Poland) at 1500× *g*, 4 °C, for 20 min. The resulting plasma was aliquoted, frozen at −75 °C, and stored until the completion of all sample collections.

The concentrations of biochemical and hormonal biomarkers associated with negative energy balance were determined in plasma. The analyzed indicators included β-hydroxybutyrate, glucose, leptin, and selected fatty acids (FAs) —C16:0, C18:0, C18:1-t9, and C18:2—which collectively served as indicators of lipolytic activity and metabolic adaptation. Plasma fatty acid composition was determined using gas chromatography with flame-ionization detection (GCMS: Agilent Technologies Inc., Wilmington, DE, USA) after lipid extraction according to the Folch method and subsequent methylation with methanolic HCl. The resulting fatty acid methyl esters (FAMEs) were identified by comparison with certified reference standards (Supelco 37 Component FAME Mix, Sigma-Aldrich, St. Louis, MO, USA). The results represent total fatty acids in plasma, as the samples were subjected to complete lipid hydrolysis prior to methylation. The concentrations of individual fatty acids were expressed as a percentage of total identified FAs.

BHBA concentrations were quantified using commercial diagnostic kits (Randox Laboratories Ltd., Crumlin, UK). Absorbance was measured with a UV–Vis spectrophotometer (Varian Inc., Palo Alto, CA, USA). Leptin (LEP) concentrations were determined using a bovine-specific enzyme-linked immunosorbent assay (ELISA) kit (EIAab, Wuhan, China). Glucose levels were measured using Randox commercial kits (Randox Laboratories Ltd., Crumlin, UK) on the same UV–Vis spectrophotometric system (Varian Inc., Palo Alto, CA, USA).

All analyses were performed in accordance with the manufacturers’ protocols, with appropriate quality control standards to ensure analytical precision and repeatability.

### 4.6. Statistical Analysis

All statistical analyses were performed using the STATISTICA software package (v.13.3; StatSoft Inc., Tulsa, OK, USA). A general linear model (GLM) with repeated measures was applied to evaluate the effects of the experimental period on the analyzed physiological, biochemical, and molecular parameters. Results are expressed as mean values ± standard error of the mean (SEM) for each stage of the study.

To assess temporal dynamics and non-linear associations among key indicators of energy balance, metabolic biomarkers, and inflammatory proteins, polynomial regression analysis was employed. The regression models were fitted to describe curvilinear trends and to visualize the relationships between energy balance and selected variables. Corresponding graphs display the modeled regression curves together with the empirical data points collected throughout the experiment, allowing for the evaluation of both fit accuracy and biological relevance.

Individual cows were included in the model as random effects to account for within-animal variability over time, while the experimental stage was treated as a fixed effect. To handle the repeated-measures structure of the data and control for potential autocorrelation across sampling points, a mixed-model approach was implemented with a first-order autoregressive covariance structure [AR(1)], which provided the best fit according to Akaike’s information criterion (AIC). Model residuals were examined for normality and homoscedasticity. Pairwise comparisons between time points were adjusted using the Tukey–Kramer post hoc correction to control the family-wise error rate. Pearson correlation coefficients were calculated to assess relationships among variables, and the corresponding *p*-values were corrected for multiple testing using the Benjamini–Hochberg false discovery rate (FDR) method, ensuring robust control of type I error.

All statistical tests were performed at a significance level of *p* ≤ 0.05, and results were considered statistically significant at this threshold. The correlation coefficient was calculated and verified at *p* < 0.01.

## 5. Conclusions

To our knowledge, this study is the first to document the concurrent regulation of interleukin-6 and plasminogen in Simmental cows during early-lactation negative energy balance. Previous investigations in Holstein-Friesian cows have evaluated IL-6 and PL individually in relation to metabolic stress; however, their coordinated regulation has not been demonstrated before. The present findings therefore provide novel evidence of a strong association between these two molecules, revealing a potential molecular interaction that integrates inflammatory and fibrinolytic responses under physiological NEB conditions. This insight advances the current understanding of how dual-purpose breeds such as Simmental achieve metabolic resilience through balanced immune–metabolic adaptation. The findings of this study provide new molecular insight into the adaptive mechanisms that enable Simmental cows to cope with the metabolic and inflammatory challenges of negative energy balance during early lactation. The onset of lactogenesis was identified as the primary trigger of spontaneous lipolysis, while the moderate increase in β-hydroxybutyrate concentrations and absence of clinical ketosis suggest that Simmental cows maintained effective lipid utilization and overall metabolic stability during this period. The concurrent elevation of interleukin-6 and plasminogen indicates activation of systemic inflammatory and fibrinolytic pathways associated with lipid mobilization and hepatic metabolism. However, the moderate and transient nature of these responses suggests a controlled physiological adjustment rather than a pathological process. Collectively, the results indicate that Simmental cows exhibit breed-associated metabolic resilience characterized by coordinated lipid metabolism and moderated cytokine activity. The observed IL-6–plasminogen relationship may reflect a molecular link between lipid mobilization and inflammatory regulation, providing potential biomarker candidates and a framework for further studies aimed at improving metabolic stability and lactational performance in dairy cattle.

## Figures and Tables

**Table 1:** Dynamics of daily milk production and basic milk composition in Simmental cows during the analyzed experimental periods and their relationship with energy balance (EB, MJNEL/day).

**Parameters**	**Time of Evaluation Relative to Calving** **(Average Day of Lactation)**					**SEM**	**Correlation (*r*)** **EB_MJNEL/d_ x**
	**SLI**	**SLII**	**SLIII**	**SLIV**	**SLV**		
	18.1	45.6	76.3	107.7	136.3		
Numbers at time points	42	42	42	42	42		
DMP (kg)	20.7 ^d^	25.6 ^bc^	30.7 ^a^	27.6 ^b^	23.4 ^b^	0.7	0.689 **
Protein	3.47 ^c^	3.44 ^c^	3.45 ^c^	3.52 ^a^	3.53 ^a^	0.07	0.245 **
Of which: WP	0.92	0.89	0.93	0.95	0.81	0.02	−0.289 **
Fat	4.21 ^b^	4.01 ^c^	3.99 ^c^	4.27 ^a^	4.31 ^a^	0.02	0.517 **
Lactose	5.06 ^c^	4.82 ^c^	4.72 ^d^	5.19 ^a^	5.24 ^a^	0.03	0.696 **
ash	0.21	0.21	0.20	0.20	0.21	0.01	-
dry matter	12.95 ^b^	12.48 ^c^	12.36 ^c^	13.18 ^a^	13.29 ^a^	0.04	0.603 **

^a, b, c, d^—*p* ≤ 0.05: different superscript letters within the same row indicate significant differences between time points; *r*—correlation for all data (**—*p* ≤ 0.01); WP—whey proteins.

**Table 2:** Dynamics of biomarker values characterizing the consequences of NEB in Simmental cows during the analyzed experimental period.

Parameters	Time of Evaluation Relative to Calving						SEM	Correlation (r) EB x
	PCP	SLI	SLII	SLIII	SLIV	SLV		
Numbers at time points	42	42	42	42	42	42		
BHBA (mmol L^−1^)	0.47 ^d^	0.52 ^d^	0.89 ^b^	1.04 ^a^	0.90 ^b^	0.69 ^c^	0.01	−0.772 **
GLU (mmol L^−1^)	2.64 ^a^	2.56 ^a^	2.31 ^b^	2.28 ^b^	2.37 ^c^	2.39 ^c^	0.01	0.579 **
LEP (ng ml^−1^)	2.42 ^c^	2.33 ^b^	2.52 ^a^	2.55 ^a^	2.49 ^a^	2.37 ^b^	0.01	−0.562 **

^a, b, c, d^—*p* ≤ 0.05: different superscript letters within the same row indicate significant differences between time points; r—correlation for all data (**—*p* ≤ 0.01); PCP—pre-calving period; EB—energy balance; BHBA—β-hydroxybutyrate; GLU—glucose, LEP—leptin.

**Table 3:** Dynamics of body condition score changes and blood concentrations of selected fatty acids in Simmental cows during the analyzed experimental periods.

Parametrs	Time of Evaluation Relative to Calving						SEM	Correlation (r) EB_MJNEL/d_ x
	PCP	SLI	SLII	SLIII	SLIV	SLV		
Numbers at time points	42	42	42	42	42	42		
BCS (point)	2.88 ^a^	2.60 ^b^	2.40 ^c^	2.19 ^d^	2.27 ^d^	2.69 ^b^	0.02	0.764 **
FAs (%):								
C16:0	22.7 ^c^	23.9 ^b^	25.7 ^a^	26.5 ^a^	25.6 ^a^	22.5 ^c^	0.1	−0.849 **
C18:0	10.4 ^c^	11.3 ^b^	11.7 ^a^	11.8 ^a^	11.1 ^a^	10.5 ^c^	0.04	−0.785 **
C18:1-t9	2.2 ^c^	2.9 ^b^	3.4 ^a^	3.6 ^a^	3.2 ^a^	2.3 ^c^	0.03	−0.824 **
C18:2	1.6 ^c^	2.3 ^a^	2.4 ^a^	2.6 ^a^	2.5 ^a^	1.8 ^c^	0.02	−0.659 **

^a, b, c, d^—*p* ≤ 0.05: different superscript letters within the same row indicate significant differences between time points; r—correlation for all data (**—*p* ≤ 0.05); PCP—pre-calving period; EB_MJNEL/d_—energy balance; BHBA—β-hydroxybutyrate; GLU—glucose, LEP—leptin, FAs—fatty acids.

**Table 4:** Relationships between blood concentrations of NEB-related biomarkers and pro-inflammatory proteins in the studied cows.

Parametrs	Time of Evaluation Relative to Calving						SEM	Correlation (r) EB_MJNEL/d_ x
	PCP	SLI	SLII	SLIII	SLIV	SLV		
Numbers at time points	42	42	42	42	42	42		
IL-6 (ng L^−1^)	87.62 ^c^	92.14 ^c^	103.82 ^a^	109.59 ^a^	105.95 ^a^	98.24 ^b^	0.61	−0.741 **
PL (ng L^−1^)	1.59 ^c^	1.65 ^c^	1.79 ^b^	2.05 ^a^	1.96 ^b^	1.81 ^b^	0.01	−0.586 **

^a, b, c^—*p* ≤ 0.05: different superscript letters within the same row indicate significant differences between time points; r—correlation for all data (**—*p* ≤ 0.01); GLU—glucose, LEP—leptin, PL—plasminogen, IL-6—interleukin-6; EB_MJNEL/d_—energy balance.

**Table 5:** Characteristics of TMR components used in feeding Simental cows during the experiment.

Nutrient Components	Period of Experience					
	PCP	I	II	III	IV	V
Crude protein (%)	15.86	15.58	16.98	16.55	16.05	12.52
Dry matter (%)	41.80	40.90	41.59	41.55	39.33	34.85
Crude fibre (%)	18.55	18.52	18.42	18.33	18.41	18.45
Crude fat (%)	2.30	2.35	2.42	2.43	2.19	2.31
Crude ash (%)	7.82	7.80	7.85	7.83	7.89	7.95
Starch (%)	21.50	22.41	22.52	22.47	21.82	21.52
Acid detergent fibre—ADF (%)	21.26	21.67	22.16	22.11	21.78	21.98
Neutral detergent fibre—NDF (%)	38.71	38.80	39.46	39.42	37.44	37.52
Physically effective NDF—peNDF (%)	29.50	29.97	34.72	34.78	31.51	30.15
JPM	20.51	24.53	28.51	27.42	22.41	20.85
BTJN (g)	2343	2349	2475	2481	2527	1698
BTJE (g)	2138	2179	2384	2366	2244	1577
Energy (MJ NEL/d):						
Requirement	142.2	148.5	159.5	158.8	157.5	112.1
Intake	141.5	146.9	156.1	155.6	155.4	113.3
Balance	−0.4	−1.6	−3.2	−3.1	−2.1	+1.2
Dry matter intake—DMI (kg/day)	22.09	22.87	22.05	22.08	23.07	23.11

**Table 6:** Components of the diet used in the feeding of Simental cows during the experiment.

Nutrient Components	Period of Experience					
	PCP	I	II	III	IV	V
Maize silage (kg)	22.0	22.0	23.0	23.5	23.5	22.5
Haylage (kg)	11.0	11.5	12.0	13.0	12.5	11.0
Ground rapeseeds (kg)	0.9	1.0	1.5	1.5	1.0	0.8
Straw (kg)	-	-	0.1	0.2	0.2	0.1
Hay (kg)	0.4	0.4	0.5	0.8	0.6	0.5
Production mix * (kg)	-	-	2.6	3.9	3.5	-

* Composition of the production mix (%): maize kernels crushed—15.5, barley—10.0, triticale—10.0, oats—16.0, Krowimix 18—2.5, ground rapeseeds—18.0, NaCl—0.3, CaCO_3_—2.3, mineral compound supplement—0.7.

## Data Availability

The original contributions presented in this study are included in the article. Further inquiries can be directed to the corresponding author.
